# Complete Genome Sequences of *Salmonella enterica* Serovar Heidelberg Strains Associated with a Multistate Food-Borne Illness Investigation

**DOI:** 10.1128/genomeA.01154-13

**Published:** 2014-06-05

**Authors:** Peter S. Evans, Yan Luo, Tim Muruvanda, Sherry Ayers, Brian Hiatt, Maria Hoffman, Shaohua Zhao, Marc W. Allard, Eric W. Brown

**Affiliations:** a Division of Microbiology, Office of Regulatory Science, Center for Food Safety and Applied Nutrition, U.S. Food and Drug Administration, College Park, Maryland, USA; b Division of Public Health Informatics and Analytics, Office of Analytics and Outreach, Center for Food Safety and Applied Nutrition, U.S. Food and Drug Administration, College Park, Maryland, USA; c Division of Animal and Food Microbiology, Office of Research, Center for Veterinary Medicine, U.S. Food and Drug Administration, Laurel, Maryland, USA; d Washington State Department of Health Public Health Laboratories, Shoreline, Washington, USA

## Abstract

Next-generation sequencing is being evaluated for use with food-borne illness investigations, especially when the outbreak strains produce patterns that cannot be discriminated from non-outbreak strains using conventional procedures. Here we report complete genome assemblies of two *Salmonella enterica* serovar Heidelberg strains with a common pulsed-field gel electrophoresis pattern isolated during an outbreak investigation.

## GENOME ANNOUNCEMENT

A multistate salmonellosis outbreak was identified using conventional typing procedures as *Salmonella enterica* subsp. *enterica* serovar Heidelberg (*S.* Heidelberg) with an indistinguishable pulsed-field gel electrophoresis (PFGE) pattern and with illness onset between 4 June 2012 and 6 May 2013 (http://www.cdc.gov/salmonella/heidelberg-02-13/index.html). Consumption of a specific brand of chicken was identified as the most likely source of the illnesses. CDC and USDA investigations focused on the source, specifically the live animal producers and slaughter facilities associated with the specific brand, but a source was not definitively identified and no product was recalled. Washington State Public Health Laboratories isolated the outbreak strain from the stool of an outbreak case patient (strain CFSAN002064, also identified as WA_EN021292) and from an unopened chicken sample collected from the patient’s home and believed to be source of the patient’s illness (CFSAN002069, also identified as WA_FL1200202). The genomic DNA from each strain was isolated from pure culture using Qiagen DNeasy blood and tissue kit (Qiagen Inc., Valencia, CA), which was then used to prepare a single 10-kb library following the Pacific Biosciences sample preparation methods for C2 chemistry. The 10-kb libraries were sequenced using Pacific Biosciences RS sequencing technology (Pacific Biosciences, Menlo Park, CA) on 8 and 10 single-molecule real-time (SMRT) cells with a 90-minute and 120-min collection protocol, achieving average genome coverage of >100× and >200×, for CFSAN002069 and CFSAN002064, respectively. *De novo* assemblies were performed on the 10-kb continuous long-read data using the SMRT Analysis (version 2.0.1, Pacific Biosciences). The genomes were annotated with the Prokaryotic Genomes Automatic Annotation Pipeline (NCBI, Bethesda, MD). Kinetic variations (KVs) in nucleotide incorporation rates (1) were used to measure epigenetic modifications. Methylation activities were deduced from the KV data and deposited into the REBASE Bacteria Genomes database (http://rebase.neb.com/rebase/arcbaclistB.html). CFSAN002064 and CFSAN002069 also were antibiotic susceptibility typed using the broth microdilution method and the National Antibiotic Resistance Monitoring System panel (2).

The case-patient isolate, CFSAN002064, was fully susceptible to all 15 antimicrobials in the panel, and the genome, when fully assembled, consisted of two contigs: one corresponding to the circular chromosome of 4,783,867 bp and the other to a circular plasmid of 37,692 bp. The food isolate, CFSAN002069, was resistant to gentamicin (GEN), streptomycin (STR), and sulfisoxazole (FIS), and the assembled genome consisted of three contigs: one corresponding to the circular chromosome of 4,783,943 bp and two corresponding to circular plasmids. One plasmid of 110,363 bp was identified as IncI containing genes conferring resistance to GEN, STR, and FIS (3), and the other smaller plasmid had 37,679 bp. The chromosome contigs of CFSAN002064 and CFSAN002069 were colinear with no rearrangements. KV data predicted the same four methylation activities in both strains. Another report will evaluate the relationships of whole genome sequences from this outbreak with non-outbreak associated strains.

### Nucleotide sequence accession numbers.

Complete genome sequences have been deposited in DDBJ/EMBL/GenBank under accession no. CP005995 and CP005994 for CFSAN002064 and CP005390, CP005389, and CP005391 for CFSAN002069.
